# Multi-omic modelling of inflammatory bowel disease with regularized canonical correlation analysis

**DOI:** 10.1371/journal.pone.0246367

**Published:** 2021-02-08

**Authors:** Lluís Revilla, Aida Mayorgas, Ana M. Corraliza, Maria C. Masamunt, Amira Metwaly, Dirk Haller, Eva Tristán, Anna Carrasco, Maria Esteve, Julian Panés, Elena Ricart, Juan J. Lozano, Azucena Salas

**Affiliations:** 1 Centro de Investigación Biomédica en Red de Enfermedades Hepática y Digestivas (CIBERehd), Barcelona, Spain; 2 Department of Gastroenterology, IDIBAPS, Hospital Clínic, Barcelona, Spain; 3 Chair of Nutrition and Immunology, Technical University of Munich, Freising-Weihenstephan, Germany; 4 ZIEL Institute for Food and Health, Technical University of Munich, Freising-Weihenstephan, Germany; 5 Department of Gastroenterology, Hospital Universitari Mútua Terrassa, Barcelona, Spain; Washington State University - Spokane, UNITED STATES

## Abstract

**Background:**

Personalized medicine requires finding relationships between variables that influence a patient’s phenotype and predicting an outcome. Sparse generalized canonical correlation analysis identifies relationships between different groups of variables. This method requires establishing a model of the expected interaction between those variables. Describing these interactions is challenging when the relationship is unknown or when there is no pre-established hypothesis. Thus, our aim was to develop a method to find the relationships between microbiome and host transcriptome data and the relevant clinical variables in a complex disease, such as Crohn’s disease.

**Results:**

We present here a method to identify interactions based on canonical correlation analysis. We show that the model is the most important factor to identify relationships between blocks using a dataset of Crohn’s disease patients with longitudinal sampling. First the analysis was tested in two previously published datasets: a glioma and a Crohn’s disease and ulcerative colitis dataset where we describe how to select the optimum parameters. Using such parameters, we analyzed our Crohn’s disease data set. We selected the model with the highest inner average variance explained to identify relationships between transcriptome, gut microbiome and clinically relevant variables. Adding the clinically relevant variables improved the average variance explained by the model compared to multiple co-inertia analysis.

**Conclusions:**

The methodology described herein provides a general framework for identifying interactions between sets of omic data and clinically relevant variables. Following this method, we found genes and microorganisms that were related to each other independently of the model, while others were specific to the model used. Thus, model selection proved crucial to finding the existing relationships in multi-omics datasets.

## Introduction

The creation of datasets from different high-throughput sequencing technologies on the same samples provides an opportunity to identify relationships between datasets and improve our understanding of diseases. This approach has been used in several diseases, such as cancer, inflammatory bowel disease (IBD) and pouchitis, among others [1–3].

IBD is comprised of Crohn’s disease (CD) and ulcerative colitis (UC). Around 4.2 million individuals suffer from IBD in Europe and North America combined [4]. The chronic inflammatory response observed suggests an interaction between host genetic factors and the intestinal microbiota. Several studies support the concept that CD arises from an exacerbated immune response against commensal gut microorganisms in genetically predisposed individuals. Nonetheless, the disease might result from imbalanced microbial composition, leading to dysbiosis [5, 6].

Understanding the contribution of the gut microbiota to CD pathogenesis and maintenance of the disease is an ongoing field of research [7–9]. These alterations could be shaped by a genetic predisposition and environmental factors (i.e., bacterial or viral infection, diet, usage of antibiotic, or the socioeconomic status) [10]. On the other hand, pouchitis refers to the inflammation of the ileal pouch, an artificial rectum surgically created out of ileal gut tissue in patients who have undergone a colectomy. One possible underlying cause of pouchitis might also be the an imbalance in the gut microbiome [11]. However, the cause-effect relation between dysbiosis and intestinal inflammatory disease remains unclear [12–14].

The most common method for analyzing the relationship between microorganisms and the gut mucosa is to sequence both the 16S rRNA gene of the microbiome and the patient’s transcriptome, respectively. Gut microbial DNA can be sequenced from feces or intestinal tissue, while human RNA is isolated from endoscopic biopsies or surgical samples. In some cases, patients are followed up for long periods and longitudinal samples can be obtained [15]. Multivariate methods are used to integrate DNA and RNA data, and therefore can identify relationships between the intestinal microbiome and the gut epithelium [8, 14, 16, 17]. Correlations, which are multivariate, are the predominant method used to find relationships between two omics datasets [7, 17–19]. A recent study revealed more significant correlations between host RNA and microbial DNA in samples from healthy controls than in patients with IBD, and suggests an “uncoupling” or breakup of these “homeostatic” correlations in diseased subjects [7]. Although their analysis used correlations, as well as univariate methods, these method do not consider confounders such as age, diet or sample localization in the gut, which could lead to false conclusions [20, 21].

Other multivariate methods provide frameworks with an unlimited number of variables involved [22, 23]. These methods summarize the variability of the datasets and select features in order to obtain loading factors for a new coordinate system where samples are represented. They summarize the largest amount of variability found among the samples’ variables [24]. Those multivariate methods are capable of summarizing several variables from the same sample. Some multivariate methods work when variables are grouped in in a block. Multi-block methods allow to analyze variables obtained from different technical origins [25–29]. These multi-block methods assume the existence of relationships between variables of the different blocks.

An example of a multi-block method is the regularized generalized canonical correlation analysis (RGCCA) which enables reducing the dimensions of an arbitrary number of blocks for data derived from the same sample [30–32]. RGCCA has already been used in the context of IBD with RNA-seq and 16S rRNA data [16]. However, it was used to select human genes and microorganism related to the inflammation predictors DUOX2 and APOA1. To our knowledge, a concrete description of the relationship between the gut’s mucosal host transcriptome and microbiome in CD using RGCCA has not been performed.

In this study, we evaluate the effect of the parameters of RGCCA on the canonical components and we identify a strategy of analysis that better explains two previously published datasets. We then used this method, as well as multiple co-inertia analysis (MCIA), to compare two datasets, our hematopoietic stem cell transplant CD dataset and an online available pouchitis dataset in order to identify interactions between microorganisms and the host transcriptome of the gut epithelium [33]. Overall, we believe that our approach constitutes an innovative method for identifying multiple relationships present in multi-omics datasets and their most relevant variables. Identifying those relevant variables will lead to discover the cross-talk between microorganisms and the host and enhance our knowledge of the inflammatory bowel disease.

## Methods

### Patients and biopsies processing

Samples from the CD dataset included in this study were from a cohort of patients with severe refractory CD undergoing hematopoietic stem cell transplant (HSCT). Patients were treated in the Department of Gastroenterology (Hospital Clínic de Barcelona–Spain–). The protocol was approved by the Catalan Transplantation Organization and by the Institutional Ethics Committee of the Hospital Clinic de Barcelona (Study Number 2012/7244). All patients provided written consent following extensive counselling. Colonic and ileal biopsies were obtained at several time points during ileocolonoscopy. Patients were followed-up for 4 years and biopsies were collected every six or twelve months after HSCT. Samples were obtained when possible from both uninvolved and involved areas. In addition, biopsies were taken from the ileum and colon regions of 19 non-IBD controls consisting of individuals with no history of IBD and who presented no significant pathological findings following endoscopic examination for colon cancer surveillance (Hospital Univesitari Mútua de Terrassa–Spain–). The protocol was approved by the Institutional Ethics Committee of the Hospital Univesitari Mútua de Terrassa (Study Number NA1651). At least one biopsy was collected and fresh-frozen at -80°C for microbial DNA extraction. The remaining biopsies were placed in RNAlater RNA Stabilization Reagent (Qiagen, Hilde, Germany) and stored at -80°C until total RNA extraction.

### Mucosal transcriptome

Total RNA from mucosal samples (HSCT CD cohort) was isolated using the RNeasy kit (Qiagen, Hilde, Germany). RNA sequencing libraries were prepared for paired-end sequencing using HighSeq-4000 platform. Later, cutadapt (version 1.7.1) was used for quality filtering and the libraries were mapped against the human reference genome using the STAR aligner (2.5.2a) with Ensembl annotation (release GRCh38.10). Read counts per gene were obtained with RSEM (version 1.2.31) as previously described [15]. Analysis was performed using R (version 3.6.1) and Bioconductor (Version 3.10) on Ubuntu 18.04. The host transcriptome was visually inspected for batch effects in PCA. Outliers and the top 10% genes using the coefficient of variation were removed (20593, with remaining 37685 genes). Data was normalized using the trimmed mean of M-values and log transformed into counts per millions using edgeR (version 3.28).

### Microbial DNA extraction from mucosal samples

Biopsies from the HSCT CD cohort were resuspended in 180 μl TET (TrisHCl 0.02M, EDTA 0.002M, Triton 1X) buffer and 20mg/ml lysozyme (Carl Roth, Quimivita, S.A.). Samples were incubated for 1h at 37°C and vortexed with 25 μl Proteinase K before incubating at 56°C for 3h. Buffer B3 (NucleoSpin Tissue Kit–Macherey-Nagel) was added followed by a heat treatment for 10 min at 70°C. After adding 100% ethanol, samples were centrifuged at 11000 x *g* for 1 min. Two washing steps were performed before eluting DNA. Concentrations and purity were checked using NanoDrop One (Thermo Fisher Scientific). Samples were immediately used or placed at -20°C for long-term storage.

### High throughput 16S ribosomal RNA (rRNA) gene sequencing

Library preparation and sequencing were performed at the Technische Universität München. Briefly, volumes of 600μL DNA stabilization solution (STRATEC biomedical) and 400μL Phenol:choloform:isoamyl alcohol (25:24:1, Sigma-Aldrich) were added to the aliquots. Microbial cells were disrupted by mechanical lysis using FastPrep-24.: Heat tratment and centrifugation were conducted after adding a cooling adaptor. Supernatatnts were treated with RNase to eliminate RNA. Total DNA was purified using gDNA columns as described in detail previously [34]. Briefly, the V3-V4 regions of 16S rRNA gene were amplified (15x15 cycles) following a previously described two-step protocol [35] using forward and reverse primers 341F-785R [36]. Purification of amplicons was performed by using the AMPure XP system (Beckmann). Next, sequencing was performed with pooled samples in paired-end modus (PE275) using an MiSeq system (Illumina, Inc.) according to the manufacturer’s instructions and 25% (v/v) PhiX standard library.

### Microbial profiling

Data analysis was carried out as previously described [37]. Processing of raw-reads was performed by using the IMNGS (version 1.0 Build 2007) pipeline based on the UPARSE approach [38]. Sequences were demultiplexed, trimmed to the first base with a quality score <3 and then paired. Sequences with less than 300 and more than 600 nucleotides and paired reads with an expected error >3 were excluded from the analysis. Trimming of the remaining reads was done by trimming 5 nucleotides from each end to avoid GC bias and non-random base composition. Operational taxonomic units (OTUs) were clustered at 97% sequence similarity. Taxonomy assignment was performed at 80% confidence level using the RDP classifier [39] and the SILVA ribosomal RNA gene database project [34]. Later the data was normalized using the same method as for RNA-seq described above. The microbiome was visually inspected for batch effects in PCA; none were found. The resulting OTUs table was normalized using edgeR (Version 3.28).

### Datasets

Table 1 shows all datasets included in the study. The glioma dataset came from diffuse intrinsic pontine glioma patients that included the host transcriptome analyzed with Agilent 44K Whole Human Genome Array G4410B and G4112F, patients copy number variation processed with the ADM-2 algorithm, and data from comparative genomic hybridization (CGH) analyzed using Mutation Surveyor software. In addition, this dataset contained information on age, localization of the tumor, sex and a numerical grading of the severity of the tumor(see Table 1) [40, 41].

**Table 1:** Summary of samples and characteristics of the datasets used.

	Glioma	CD/UC	HSCT CD	Pouchitis
**Samples (non-disease/diseased)**	0/53	33/26	51/107	0/255
**Sex (female/male)**	28/25	42/17	22/15	101/102
**Location**	Cort: 20	Ileum:30	Ileum: 48	Pouch: 59
	Dipg: 22	Colon:29	Colon: 108	PPI: 196
	Midl: 11		Unknown: 2	
**SES-CD local (mean (min-max))**	NA		2.15 (0–12)	NA
**CDAI mean (min-max)**	NA		120 (0–450)	NA
**Age at diagnostic (<16/16<x<40/x>40 years)**			7/11/0	
**Years of disease: mean (min-max)**			14 (8–28)	

PPI: pre-pouch ileum. Cort: supratentorial, midl: central nuclei, dipg: brain stem. NA not applicable; an empty cell signifies unknown. Only the HSCT CD dataset was generated by the authors, all the other datasets were previously made publicly available.

An IBD-related dataset was obtained from Prof. Dr. Rosentiel and Prof. Dr. Robert Häsler. It included samples from the terminal ileum and sigma from CD, UC, infectious disease-controls and healthy controls (see Table 1) [7]. The provided data included location, gender, location, age, and the status (inflamed or non-inflamed) of the region from which the biopsy was taken. The HSCT CD cohort involved 158 samples (both host RNA and microbial DNA) from 18 CD patients undergoing HSCT in our center and 19 non-IBD controls (Table 1) [15]. In addition to the samples, clinical information such as age, sex, treatment, years since disease diagnosis, prior surgery, location of the biopsies, segmental simple endoscopic score for Crohn’s disease (SES-CD), time of the HSCT and response to treatment were collected. A previously published dataset from a pouchitis study was also analyzed (Table 1) [33]. A total of 255 samples from 203 patients were used containing data for both host transcriptome and microbiome. This dataset included identifiers for the patients, whether the sample was from the pre-pouch ileum or from the pouch, the sex, the outcome of the procedure and an inflammatory severity score ISCORE. The pouch ileum might be inflamed or not.

### Integration

Sparse regularized generalized canonical correlation analysis (SRGCCA), implemented in RGCCA package (version 2.12), was used for this integration analysis [42]. This variation of the RGCCA method is better suited for biological data with sparsity such as the results obtained by RNA sequencing. The scheme used to add the different canonical components was the centroid scheme, which allows one to determine the positive and negative related variables. The regularization parameters used were those suggested by the tau.estimate, which is a compromise between correlation and covariance also known as Schäfer’s method [43]. When looking for the covariance from phenotypic categorical variables in order to maximize the covariance instead of the correlation 1 was used for regularization.

Numeric values from the same assay were set on the same block. Relevant clinical variables were grouped in one block unless otherwise indicated. Categorical data was encoded as binary (dummy) variables for each factor, where 0 indicates not present and 1 indicates present omitting one level. Each block was standardized to zero mean and unit variances, and then divided by the square root of the number of variables of the block with the function scale2.

MCIA was also performed on the CD/UC, CD and pouchitis dataset using only the experimental data [28]. RGCCA was compared to MCIA by examining the area under the curve (AUC) of both methods when classifying localization on the first component of the shared latent space of MCIA and the first component of the host transcriptome on the RGCCA method.

### Parameter testing

The sparse canonical correlation analysis involved three parameters besides the input data: the regularization parameter (tau) the model and the scheme. To evaluate the effect of each parameter, the one being tested was changed while keeping constant all the others. This model included weights indicating the relationship between the blocks. These parameters were tested on the glioma dataset and on the CD/UC dataset.

All models were analyzed using weights from 0 to 1 by 0.1 intervals in the relationship between blocks. These weights indicate the strength of the relationship between the variables of two blocks, the higher it is, the stronger is the relationship between the variables. To test the effect of the model, all combinations of weights were analyzed. The indicators of methods quality consist of the inner average variance explained (AVE) the outer AVE and the AVE of each block. The inner AVE is defined by how well the components of each block correlate with one other [31]. The outer AVE is defined by how well the variables of a block correlate with the component for all of the blocks. As we were interested in discovering the relationships between blocks, the inner AVE was used to select the best model, the higher the inner AVE is, the better the model.

The scheme controls how the different correlations of the canonical components are summarized. The three schemes available (horst, centroid and factorial) are compared using a simple model regarding their inner AVE and the selected genes.

Tau was tested on the glioma and the CD/UC dataset between the minimum accepted value and 1 for each block.

Models were validated using 1000 bootstraps with resampling to assess the stability of the inner and outer AVE.

### Models used

Different models were tested for the integration of the data from the CD or the pouchitis dataset. The first model, model 0, used only two blocks, the microbiome and the host transcriptome data with interaction between them, but with no within interactions (Model not shown).

The second family of models (models 1, 1.1 and 1.2), family 1, in addition to the microbiome and host transcriptome data, included those variables we considered clinically relevant variables including some that were related to disease activity. This model was explored because it takes into account already known information that could help reveal relevant relationships. For instance, the HSCT CD dataset included the following variables: patient ID, sex, age, age at diagnosis, previous surgery, current treatment, time after HSCT and location of the sample. Including these variables could potentially help to reveal a relationship that changes with patient’s characteristics, time and location.

The last family of models (models 2, 2.1, 2.2 and 2.3), family 2, used the same information as that for family 1 models, but grouped the clinical variables into three blocks, one for demographics, one for time-related variables and one for variables related to localization of the sample. Although, this family of models is more complex than family 1 the relationships found can potentially occur independently of time, clinical variables and location, thus revealing other relationships that could not be identified using the family 1 models. All models can be found on S1 Data. Models 1 to 2.3 were modeled to utilize known, clinically relevant variables with the host transcriptome and microbiome data available.

With the glioma dataset, the microbiome block was replaced by the CGH block. In addition to the previously mentioned models, the glioma dataset was also analyzed considering all the variables from the different blocks as a single entity, which is known as a superblock [44]. A superblock is a block created with all the variables on the system usually connected with each individual block of the system being analyzed.

Only the models in which all the blocks were part of a single connected network were analyzed, thus, 31 of all possible models were filtered out. For models 1 to 2.3, all the combinations of different weights on the model matrix were analyzed. First weights 0, 0.5 and 1 were used to select the model with the highest inner AVE. To further describe the interactions of models 1.1 and 2.1, different weights from 0 to 1 by 0.1 intervals were tested; the best model of each family resulted from model 1.2 and 2.2, respectively. By taking into account a direct interaction between the microbiome and the host transcriptome we could confirm whether the results of model 2.2 had improved in model.

## Results

### Parameters on the glioma dataset

We first determine the best strategy to obtain the right values of the parameters on SRGCCA using the glioma dataset. This was the dataset originally used to develop and test the SRGCCA method [39]. By parameters we mean the scheme used, the regularization effect, and the models as constructed by weights, all of which can affect the final solution of the SRGCCA (See Parameters testing in Methods).

**Fig 1:**
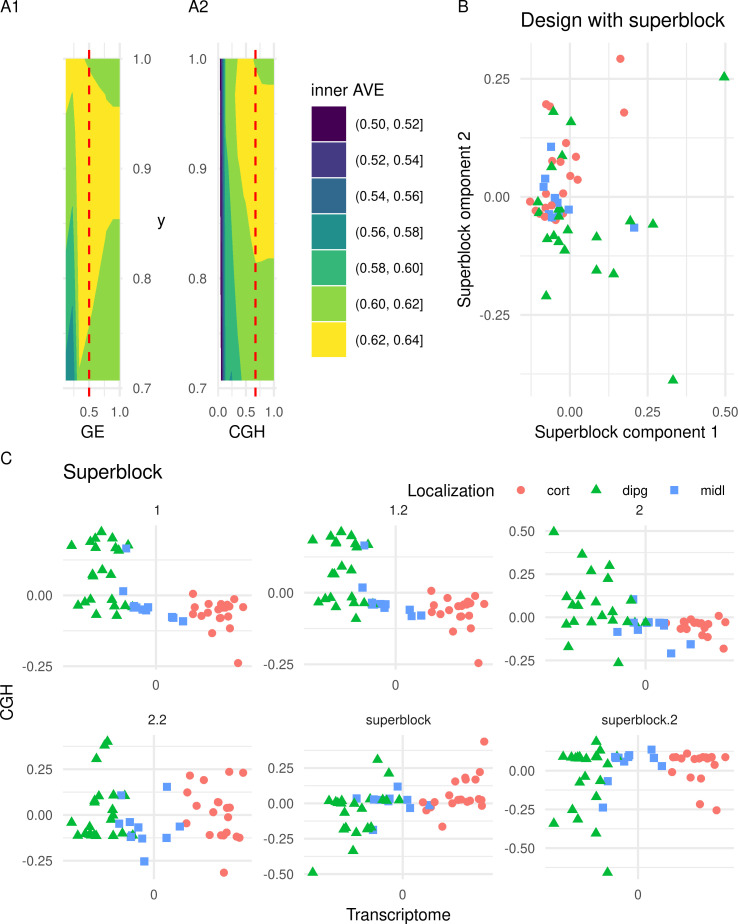
Analysis of the parameters on the glioma dataset. A1 and A2: A contour plot of the median of the inner AVEresult of an SRGCCA with different tau values for each block (GE, gene expression of the host transcriptome, CGH (comparative genomic hybridization) for the copy number variation and y for the location). Higher tau normally increases the inner AVE, Schäfer’s approximation is marked with the red vertical line. B: First two dimensions of the superblock on the glioma dataset. The first two components of the superblock within the best model, according to the inner AVE from the glioma dataset. C: First dimensions of the host transcriptome and the CGH block of models on the glioma dataset are represented. Comparison of the different models by visualizing the first components of the host transcriptome gene expression (GE) and the copy number variation (CGH) blocks from the glioma dataset. Each point represents a sample (colored by location). Cort: supratentorial, dipg: brain stem, midl: central nuclei.

Tau controls the number of variables selected from each block, regulating the stringency of the model. Tau can be estimated using Schäfer’s method [43], which tries to balance both the correlation and the covariance for selecting the variables of the block. When estimated by this method, the tau provides a good intermediate solution for numeric variables. For those blocks that encode categorical variables as numeric values, the covariance of the block with the other block is the only relevant meaning; thus, a tau value of 1 is more appropriate although several values were explored. The effect of tau on the inner AVE is shown in Fig 1A, where usually an increase on tau increases the inner AVE as well, although Schäfer’s method provided result is close to the optimum value.

All the weights between 0 and 1 (by 0.1 intervals) in the glioma dataset were analyzed using all three schemes: horst, centroid and factorial. The horst and the centroid scheme were similar while the factorial resulted in the most different AVE values (see S1 Data). The centroid scheme takes into account all the relationship regardless of the canonical correlation sign. This, together with its similarity to horst scheme, prompted its selection as the best scheme.

The three blocks with the best tau and the centroid scheme were analyzed by changing the weights between 0 and 1 by 0.1 intervals. According to the inner AVE, the best model was the one in which the weights (1) between the host transcriptome and location, (2) the host transcriptome and the CGH, and (3) the CGH block were linked to variables related to the location with weights of 1, 0.1 and 0.1, respectively.

When we added a superblock to the data, there was an increase of 0.01 on the inner AVE of the model (See Methods section Models used and [44]). The model with the superblock that explained most of the variance was that in which the weights of the interaction within (1) the host transcriptome, (2) between the superblock and the CGH, (3) between the host transcriptome and the localization, and (4) between CGH and the host transcriptome were 1, 1, 1 and 1/3, respectively. To see if the superblock could classify the sample by location, we plotted the first two components of the superblock (see Fig 1B). We can clearly see that they do not classify the samples according to the location of the tumor, which is known to affect the tumor phenotype [40].

Adding one block containing the age of the patient and the severity of the tumor to the model, decreased the inner AVE. The best model with these blocks, according to the inner AVE, was that in which the interactions (1) within the host transcriptome, (2) between the host transcriptome and the localization, (3) between the host transcriptome and(4) the CGH and between the CGH and the other variables were 1, 1, 1/3 and 1/3, respectively (see S2 Data, Glioma sheet). The first components of each model can be seen in Fig 1C. We can observe on the figure, the strong dependency between gene expression and location since the first model while the weaker relationship with the CGH assay [40]. On the other hand, the major difference is the dispersion on the CGH component on each model.

As the model with a superblock did not help explain the relationships between blocks, we decided not to apply it to the other datasets. The scheme selected was the centroid, which takes the absolute value of the relation between components. These parameters were used for further analysis on the CD/UC, the CD and pouchitis datasets.

### Parameters on the CD/UC dataset

After an exploratory analysis of the parameters on the glioma dataset, we analyzed the CD/UC dataset, which was similar to our CD dataset and include information on both the host transcriptomics and bacterial genomics. These data were obtained using the same sequencing techniques from endoscopic biopsies.

In this dataset, the parameter tau behaved slightly differently than with the previous dataset but the value from the Schäfer’s method for tau was close to the best value (see S1 Fig).

**Fig 2:**
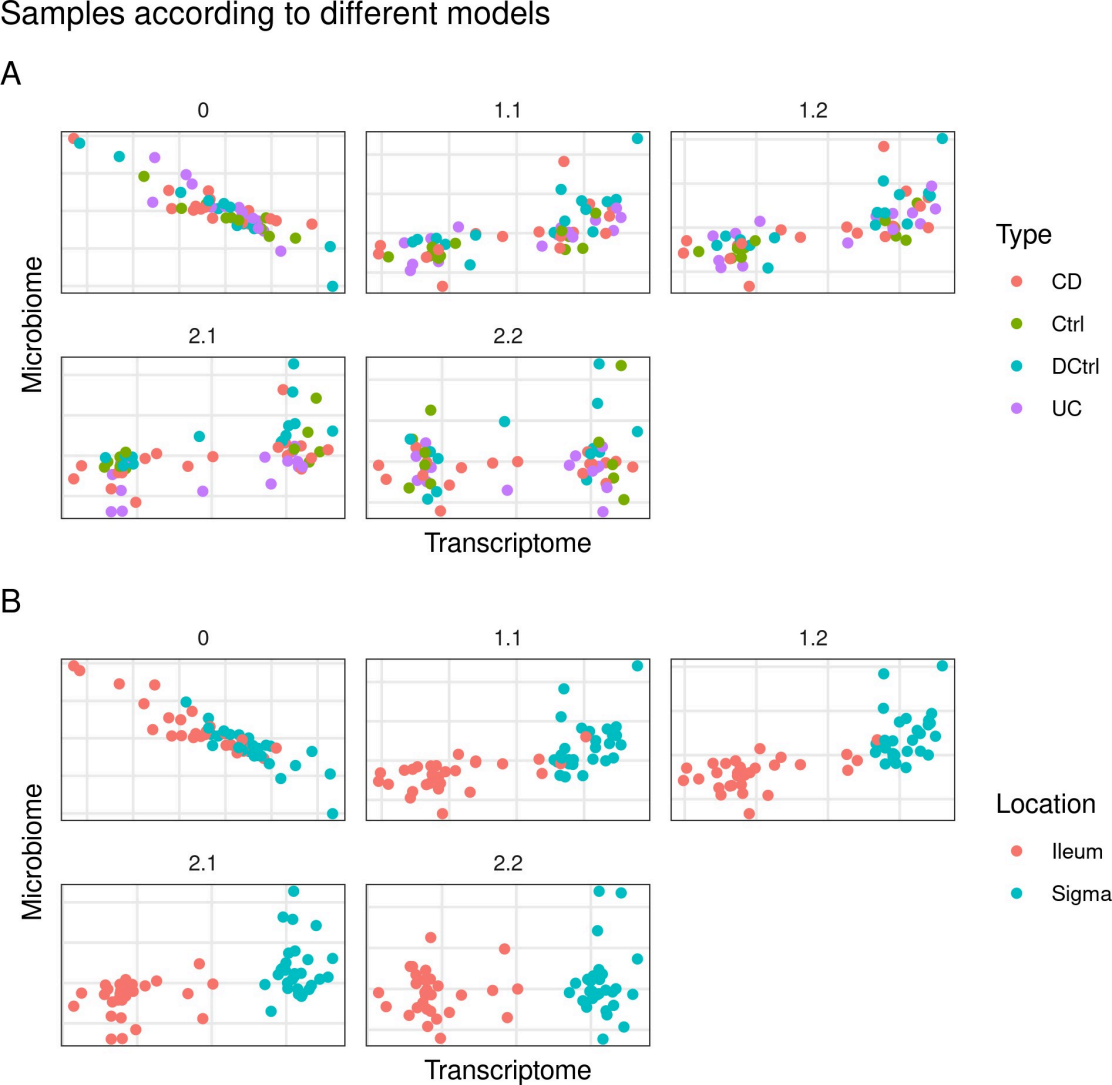
First dimensions of the host transcriptome and the microbiome block of models on the Crohn’s disease ulcerative colitis/ dataset. Comparison of the models that better explained the interaction between the microbiome and the host transcriptome data on the CD/UC dataset. Each point represents a sample colored according to a characteristic: A) samples are colored by disease type, CD Crohn’s disease, Ctrl, control; DCtrl diseased control, inflamed but not from IBD patients, UC ulcerative Colitis; and B, by location, colon or ileum, on the first components of the host transcriptome and the microbiome. Better models separate samples by tissue location using the host transcriptome component.

In contrast to the glioma dataset, the model with the highest inner AVE was model 1.2 (S2 Data). Model 2.2 has a relationship of 0.1 between microbiome and the host transcriptome and of 1 between the location and the host transcriptome. The microbiome block is also related by a factor of 0.1 with the demographic block and of 1 with the time block. Lastly the time and the demographic block are related by a factor of a 0.1. In either case the family 1 and family 2 models can correctly separate by sample location (colon or ileum) but not by disease type (see Fig 2) or inflammation status (data not shown).

### Analyzing the models on the HSCT CD and pouchitis datasets

**Fig 3:**
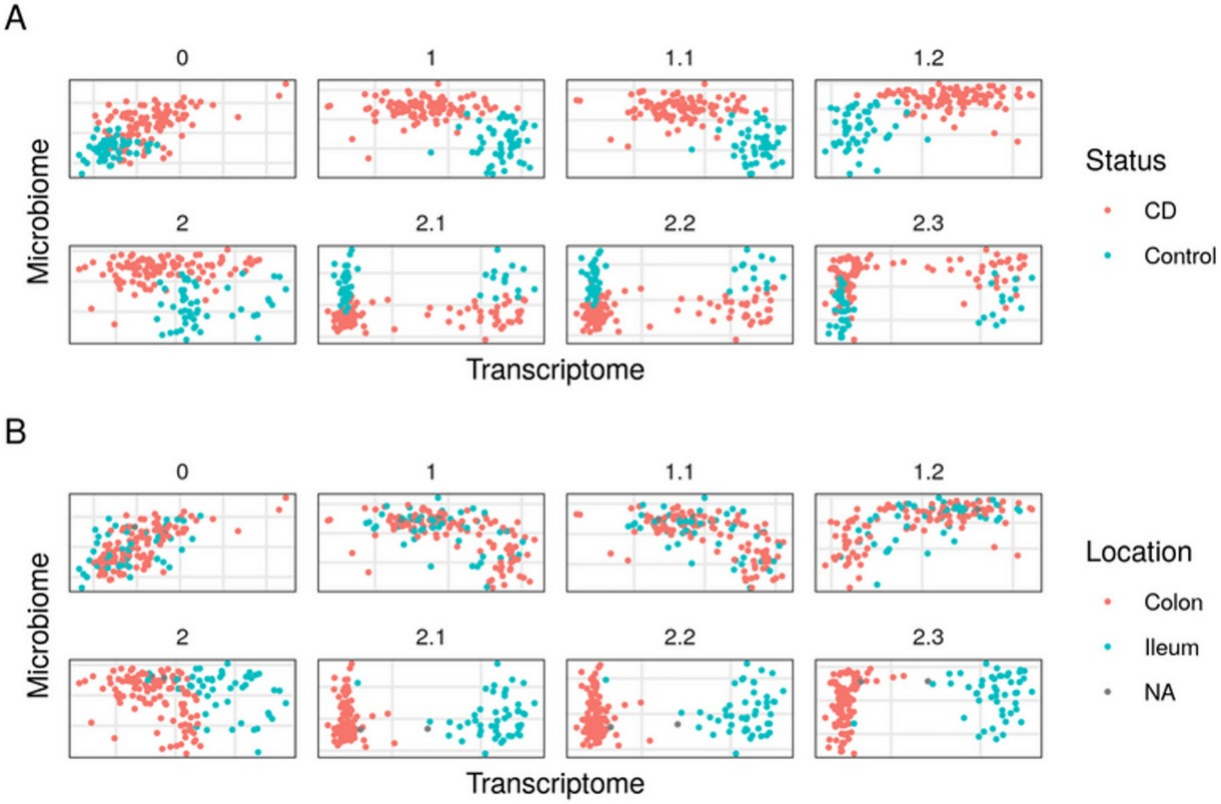
First dimensions of the host transcriptome and the microbiome block of models on the hematopoietic stem cell transplant Crohn’s disease dataset. Comparison of the models that better explained the interaction between the microbiome and the host transcriptome data on the HSCT CD dataset. Each point represents a sample (colored by disease status): A, non-CD (Control) or CD; and B, by location, colon or ileum, on the first components of the host transcriptome and the microbiome. Better models separate samples by tissue location by the host transcriptome component and the diseased and controls samples by the microbiome component.

Having established the best parameters for analyzing a related IBD dataset, we studied our HSCT CD dataset using SRGCCA. Model 1.2 had the highest inner AVE of the family 1 model. A search for the highest inner AVE within the family 2 models resulted in model 2.2 (S2 Data). This model revealed a direct relationship between the host transcriptome and the location-related variables, while the microbiome was associated with the demographic and location-related variables (see Fig 3 and S2 Data). Overall, we see that the relationships in the model affected the distribution of samples on the components of both the host transcriptome and the microbiome.

**Fig 4:**
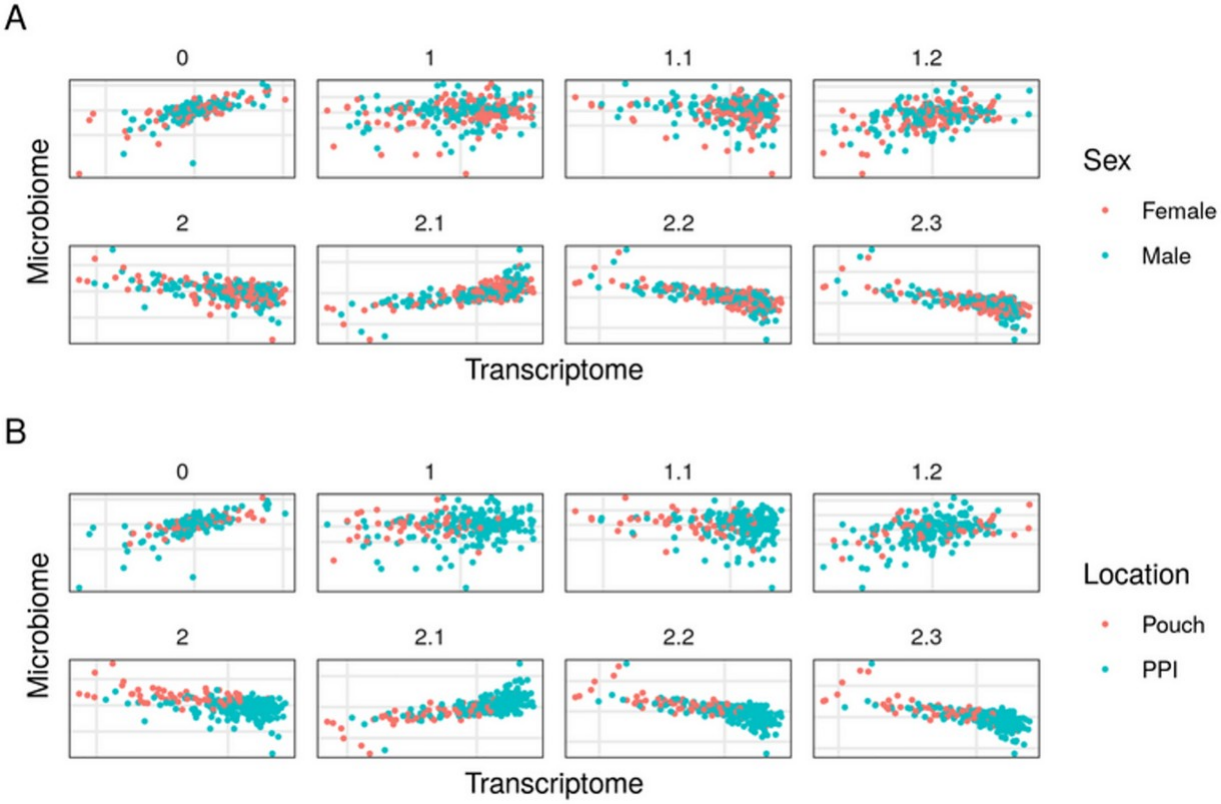
First dimensions of the host transcriptome and the microbiome block of models on the pouchitis dataset. Comparison of the models vis-à-vis on the pouchitis dataset by the first component of the host transcriptome and the microbiome from the HSCT CD dataset. Each point represents a sample colored by sex (A), where females are in red and males in blue, and by location (B), where the pouch is the red, and PPI is the pre-pouch ileum. The samples do not show a sex-specific pattern but on the best models the host transcriptome partially separates pouch and pre-pouch ileum samples.

Finally, we used another related cohort to confirm the applicability of SRGCCA to an independent dataset (see Fig 4). Model 1.2 had the highest inner AVE. A search for the highest inner AVE among the family 2 models resulted in model 2.2, although it did not have a higher inner AVE than model 1.2. Moreover, no direct relationship between the host transcriptome and the clinically relevant variables was apparent (S2 Data). Family 2 models better stratified the samples by location (pouch vs pre-pouch) than did those of family 1. Nonetheless, they were separated by location-related variables in some models, albeit not as clearly as with the HSCT CD dataset. This might indicate that while sex does not affect the interaction, the location-related variables do affect the pouchitis.

Of all these models, as described above, the best according to the inner AVE on the HSCT CD dataset was model 2.2. This model explained known differences between the host transcriptome gut regions [15]. The microbiome separated the samples by disease status, indicating that it was highly relevant for the relationship with the host transcriptome.

Using the HSCT CD dataset we also looked for the best model using a single block for the clinically relevant variables, following the family model 1 structure. The model from family 1 models with the highest AVE was that in which the transcriptomics was related to the phenotype by 0.1, while the microbiome was related to the clinically relevant variables by 1. This model revealed that the relationship between the microbiome and the clinically relevant variables carried more weight than that between the clinically relevant variables and the transcriptomics on the HSCT CD dataset.

In addition, the host transcriptome was related to location-dependent variables by a weight of 1, while the microbiome was related to demographic variables, and to location related variables, by a weight of 1 and 0.5, respectively. Demographic variables were also linked by 1 to the time variables block (see S2 Data, HSCT_CD sheet).

The interaction of genes within the host transcriptome was also analyzed on the HSCT CD dataset. Adding this interaction increased the inner AVE score between 0.10 and 0.03 depending on the model. However, it was not deemed important to find the relationships between the host transcriptome and the microbiome and thus was not compared between datasets.

**Fig 5:**
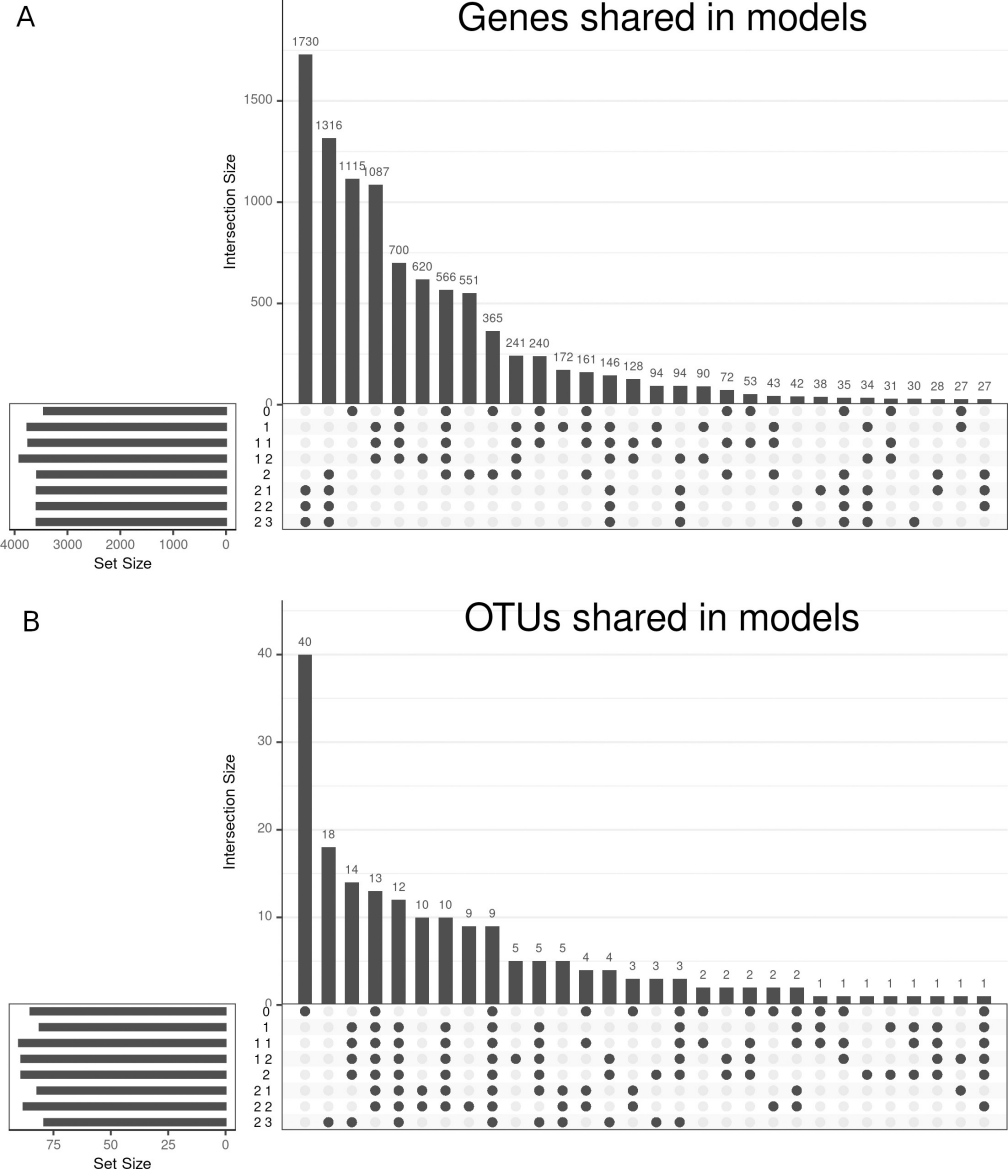
UpSet plot of the of the models on the hematopoietic stem cell transplant Crohn’s disease dataset. The heights of the bars represent the genes (A) or OTUs (B) shared between the models selected by the points; 30 intersections are shown.

Genes selected by SRGCCA as related to the microbiome in our HSCT CD dataset were different between the family 1 and 2 models (see Fig 5A), suggesting that the relationship between microorganisms and genes is independently influenced by location, time and demographic-related variables. The influence of the microbiome remained constant as indicated by the high number of OTUs shared between family 1 and 2 models suggesting that previously observed differences might have been due to covariates since the microorganisms identified by multiple models remained unchanged (Fig 5B).

### Comparison of models

**Fig 6:**
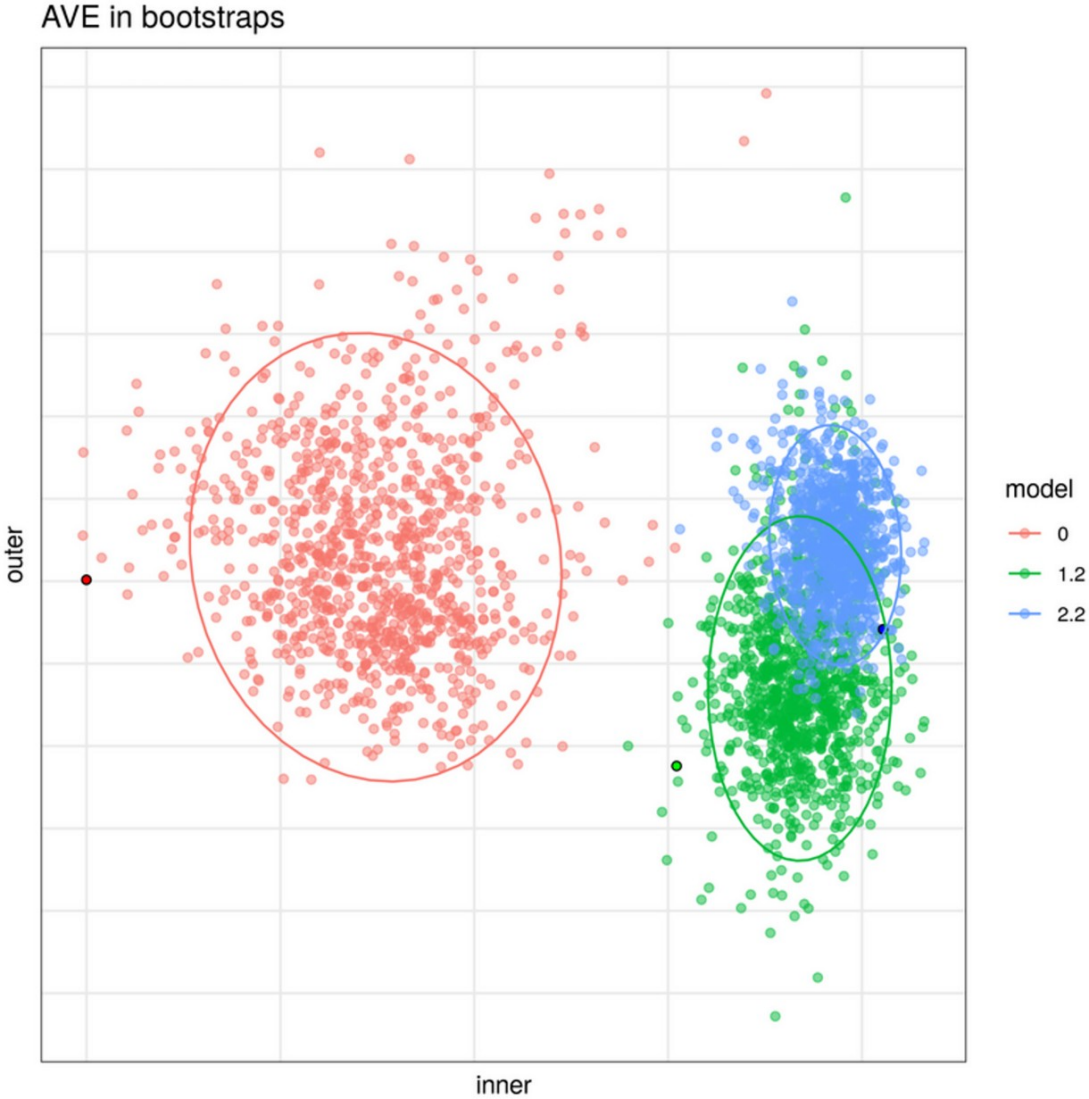
Bootstrap results of three models on the hematopoietic stem cell transplant Crohn’s disease dataset. Variance of AVE using the same samples on three models with the HSCT CD dataset. Each point shows the AVE for each analysis performed. The brighter colors reflect the result of this model on the original data (including all samples). Dispersion on the bootstrapped samples is reduced as a model more accurately represents the relationships present on the dataset.

As expected, when analyzing the same dataset with different models the output results in different relevant variables. In order to analyze the accuracy of the models, one thousand bootstraps were used to integrate the data from the HSCT CD dataset (Fig 4 and Table 2). Each model had its own dispersion on the same bootstrapped samples (Fig 6). The lower the dispersion, the more robust the model was to different conditions than in the initial testing.

**Table 2:** Bootstrapped mean and standard deviation of inner and outer AVE values on the HSCT CD dataset.

Model	AVE	Mean	Sd
0	inner	0,550	0,0469
1.2	inner	0,768	0,0223
**2.2**	**inner**	**0,785**	**0,0163**
0	outer	0,104	0,0132
1.2	outer	0,088	0,0106
**2.2**	**outer**	**0,105**	**0,0069**

The best models according to the mean are shown in bold.

Model 2.2 had both higher inner and outer AVE mean values and less standard deviation (Fig 4 and Table 2). This indicates that it was more robust than the other models, regardless of the input data.

The bootstrap analysis of the one thousand bootstraps on the pouchitis dataset showed that model 1.2 had the highest mean inner AVE, while model 0 had the highest mean outer AVE (Table 3). Overall, model 1.2 was considered the most robust.

**Table 3:** Bootstrapped mean and standard deviation of inner and outer AVE values on the pouchitis dataset.

Model	AVE	Mean	Sd
0	inner	0,448	0,0811
**1.2**	**inner**	**0,820**	**0,0457**
2.2	inner	0,767	0,0332
**0**	**outer**	**0,140**	**0,0087**
1.2	outer	0,120	0,0227
2.2	outer	0,134	0,0085

The models with the higher mean AVE values are shown in bold.

The models with the highest inner AVE were more robust to different data, which indicates that they can be applied more generally and not solely to these samples.

### Comparison of methods

We have seen that this method provides robust models of the interactions on the datasets. However, given the many methods available for integration multiple omics, we sought to determine how these methods would perform compared to other existing approaches. In particular, we ran a comparison with MCIA, which is a newer method that requires less parameters while still being conceptually similar to SRGCCA.

**Fig 7:**
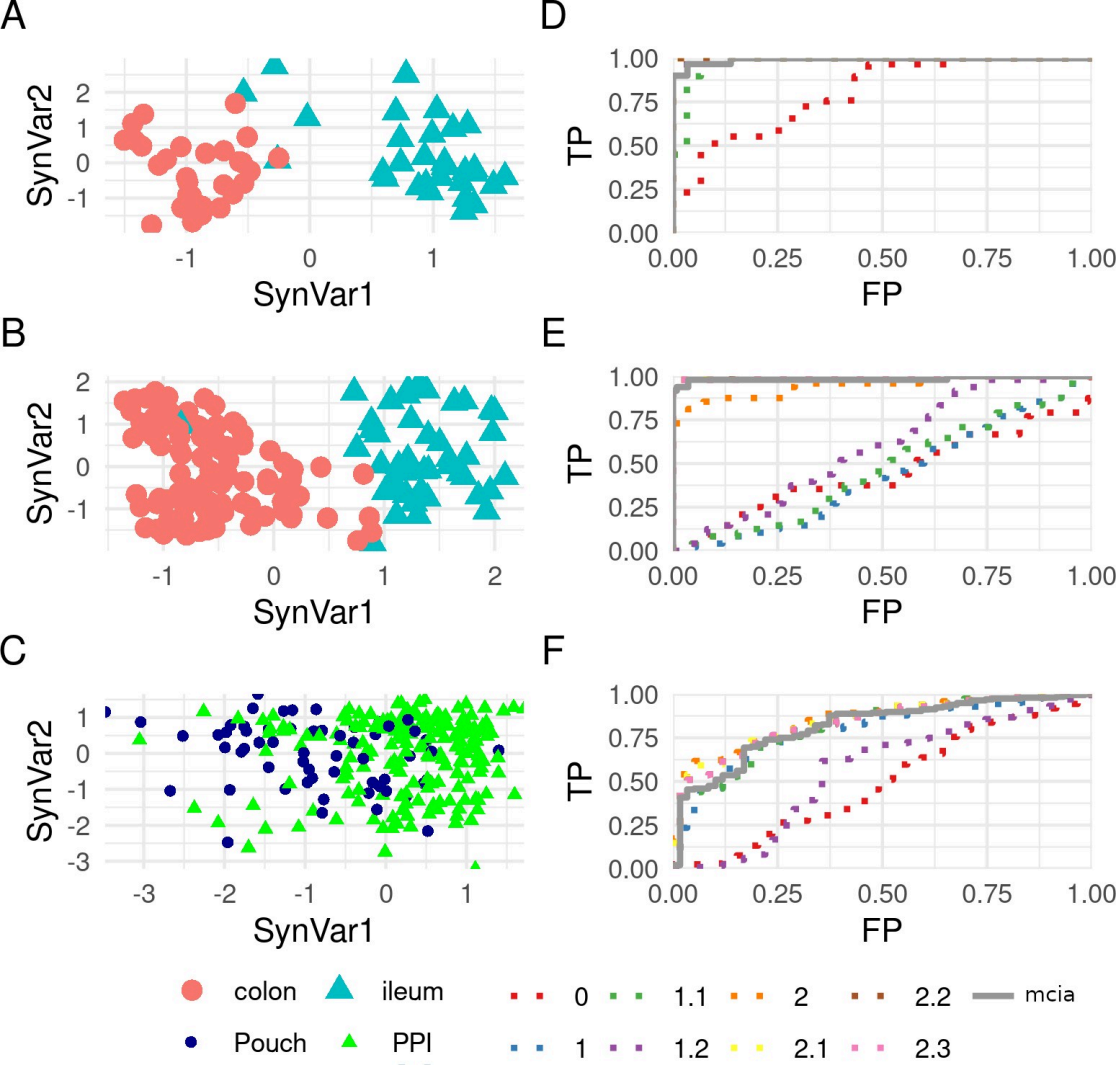
Multiple co-inertia analysis and area under the curve for the location of the Crohn’s disease/ulcerative colitis, the hematopoietic stem cell transplant Crohn’s disease and pouchitis dataset. A, B, C plots are the results of applying multiple co-inertia analysis (MCIA) where the horizontal and vertical axis represent the synthetic variable 1 and 2 respectively. D, E, F plots are the area under the curve (AUC) for all the methods applied on this dataset. The first row (A, D) is the analysis of CD/UC dataset, the second one (B, E) the HSCT CD dataset, and the third one (C, F) the pouchitis dataset.

Applying MCIA to the CD/UC, HSCT CD and pouchitis datasets produced similar distribution on the synthetic space compared to our method (Fig 7). This method was able to classify the samples by their location on the first component in a manner similar to our own method with the first transcriptomic component. On the pouchitis dataset neither method could separate the samples by location while MCIA did worse than our best model according to the AUC. In all three datasets the best model outperformed MCIA when classifying the samples according to their location (Fig 7), with the greater difference involving the pouchitis dataset (data not shown).

## Discussion

This study provides a framework for identifying interactions between blocks of data, a step towards understanding biological relationships between datasets or between datasets and other particularly relevant variables. First, we studied the parameters’ influence on a glioma and CD/UC dataset, adjusting their values and testing how generalizable they are. Then, we developed a method to find the best model for the relationships between blocks. Lastly, we validated the method in two independent datasets.

We explored the regularization of the blocks on two previously published datasets from glioma and IBD patients. The regularization of a block modulates how many variables are selected and whether correlation or covariance have to be used when looking for the canonical correlation with other blocks [28, 30]. A tau value of 1 allowed us to select all variables, which maximized their covariance. On blocks that included only clinically relevant categorical variables, regularization must be equal to 1, since correlations with categorical variables have a different meaning. As the host transcriptome and microbiome blocks contain many variables, a shrinkage parameter closer to 0 was expected, as observed with the glioma and the CD/UC datasets. In addition, estimating tau for the quantitative blocks resulted in higher inner AVE scores since the quantitative variables that contributed most to the data variation were selected.

Based on the regularization obtained, we explored different schemes of integration on the glioma dataset. The resulting canonical components of the centroid and horst schemes did differ in some models. In fact, the canonical correlations between blocks were likely positive, making the differences between these two schemes unobservable. The centroid scheme was selected to analyze the CD and the pouchitis datasets, since canonical correlations are not always positive.

Independently of the scheme involved, a superblock not only aids in interpretation, but also helps account for the possibility of interactions between variables of the same block. The increase observed in the inner AVE may have stemmed from the interaction between variables of the same block. However, such an interpretation is not as clear as with blocks generated by a single assay or from closely related variables [30]. The superblock, which is used for redundancy analysis, did not help in terms of grouping different samples [44]. Moreover, if the goal of the model is to accurately represent the system under study, the superblock is not necessary, regardless of the assistance it provides in improving the inner AVE.

The superblock is usually related to all the other blocks. Typically, a weight of 1 is used to indicate a direct relationship between two blocks. Modifying the weights of the model influenced the result by changing AVE scores and the variables selected from each block. The highest inner AVE score was not defined by the highest weights on all the relationships.

The weights of the models represent how much one block interacts with another if the interactions are linear, an assumption of any canonical correlation [31]. In such cases, the weights are representative of the interactions between blocks.

The weights define the relationships between blocks in SRGCCA, which together determine the model of the components. Other methods like MCIA and joint and individual variation-explained (JIVE) assume a common relationship between all components, which results in a common space for the samples [27, 28]. This difference is crucial for exploring the role of the components; for example, in our manuscript each model represents the same system with different interactions and assumptions. Comparing different models after the SRGCCA led to explanations for different aspects of the same system. Here, we also show that compared to our method, MCIA can results in similar samples’ classification on a latent space. However, it was not always as good as was evident by the AUC when classifying the samples by location. In addition, the interpretation of the MCIA was not as straight forward as with SRGCCA. Furthermore, with our method the observed classification of the samples according to their location can be directly attributed to the host transcriptome while with MCIA that effect could result from either the host transcriptome or the microbiome.

Looking at the glioma data, the best model according to the inner AVE was that with the superblock. As previously explained, this model might represent the hierarchical relationships present in the data. However, the superblock did not provide more interpretable results in the glioma dataset.

In the glioma dataset, the model lacking the superblock but with the highest inner AVE indicated that the localization of a tumor influences the host transcriptome to a greater degree than the copy number variations, if the relationships are linear. Adding supplementary information on the samples’ localization did not increase the inner AVE, suggesting that there was a high dependence between localization and the tumor host transcriptome.

Interactions within the host transcriptome usually increase the inner AVE of the models. With the CD and the pouchitis datasets, self-interaction increased the inner AVE, as well as the selected features, except in models 0 to 1.2 in the CD data set. This suggests that the interactions within the same omic block become relevant if the model does not take into account the interaction between other clinically relevant variables. If other relevant variables are included, then the effect of this interaction is significantly less.

Model 0 looked for direct relationships between the microbiome and the host transcriptome. Confounders that influence both host transcriptome and microbiome, such as age or the localization and inflammation status, were not taken into account in this model. This is due to the fact that they can bias the relations found with this model [45]. Nonetheless, this model was capable of grouping the samples of the CD dataset according to their disease status, though this was not true of the pouchitis dataset.

Family 1 models use three blocks, including one for clinically important information about the samples. This new block was added to avoid biasing the integration by known factors of the samples such as sex, or location. In the best model of this family, the microbiome block had a weak relationship with the host transcriptome. This weak relationship was possibly an indicative of not lineal relations. If the relationships were not lineal, then they could not be fully identified by RGCCA [31]. Another possibility is that the microbiome was related to other variables not included on the dataset.

Finally, family 2 models, compared to those of family 1, were designed to explain the relationship between the microbiome and host transcriptome, allowing for the presence of independent interactions with location, age and other demographic-related variables. In family 1 models all the relevant variables were mixed together. In order to allow for such interactions, unrelated variables were separated in different blocks.

In the HSCT CD dataset, a cursory analysis confirmed that the genes selected by SRGCCA with model 2.2 were related to the sample location [15]. The selected microorganisms previously linked to CD dysbiosis were *Faecalibacterium sp*. and *Bacteroides sp*. (see S3 Data) [46]. This suggests that the variables selected were relevant for their role in both the tissue and the disease. Thus, the genes and microorganisms that have significant relationships were likely to be present in this context.

There are several previously known interactions between the variables collected on the multiple datasets. For instance the butyrate produced by the microbiome affects the state of the epithelial cells, implying a relationship between the microbiome and the host transcriptome [47]. It is also known that the microbiome changes along the gastrointestinal tract; thus, the microbiome and host transcriptome blocks must be connected [48]. Moreover, the microbiome is influenced by diet, which would imply a relationship between demographics and the microbiome [49]. In addition, there are some studies that observe changes over time, with perhaps additional links to changes in diet. With our method we could a connection between all of these blocks.

In the pouchitis dataset, model 1.2 captured a greater degree of variance than model 2.2, contrary to the results obtained with the HSCT CD dataset. This might be because potentially important variables, such as age, were lacking and possibly because the model was confounded. In addition, we could not make direct comparisons with the HSCT CD dataset as it did not include non-diseased samples although it did include non-inflamed samples. This is due to the fact that the model differentiates by subgroups of patients instead of by a distinct relationship between healthy and diseased samples.

The findings of this study have to be assessed in light of certain limitations. RGCCA cannot describe a causal relationship or the mechanisms underlying the relationships between RNA transcriptomics and the microbiome. However, models for RGCCA can be used to select variables for further studies and experiments in order to validate these relationships. This method has been implemented in an R package, called inteRmodel, which can be found at https://github.om/llrs/inteRmodel/. This package implements the methodology described in this manuscript and also incorporate some help functions for the analysis.

When examining an interaction within a block, we only assumed the existence of an interaction within the host transcriptome. However, it must be noted that microorganisms create communities for which the interactions of several microorganisms is essential and we did not consider interaction within the microbiome in the present study [50]. Knowing how microbial communities rise and interact remains an open question that could affect any interpretation of the results [50, 51]. In addition, the taxonomy imputation can be biased by the copy number variation of the 16S rRNA present on the microbiome. This problem has not yet been solved, and the workflow used could over-estimate the abundance of some taxonomies [52].

In the present study, as we did not use a simulated data set with known relationships between blocks, we could not assess the specificity or sensitivity of our approach. In addition, we could not confirm by further analysis and experiments whether the selected variables were necessary to start or maintain CD or pouchitis.

## Conclusions

RGCCA is a powerful integration tool. We have shown that the model is the most important parameter when selecting variables. The weights of the model represent the strengths of the relationships between blocks. Here we propose a robust methodology implemented with inteRmodel, to identify the best models guided by the inner AVE when there is no prior knowledge of the existing relationship.

This method can identify relationships in complex systems such as Crohn’s disease by taking into account the interactions between the microbiome, host transcriptome and the relevant clinical variables. The resulting analysis can improve our understanding of the biological relationships between different omics datasets and other relevant (clinical) variables.

## Supporting information

S1 Data Schemes types and AVE on the glioma dataset. (XLSX)

S2 Data Models design representations. (XLSX)

S3 Data Variables selected by the model 2.2 on the HSCT CD dataset. (XLSX)

S1 Fig Tau effect on the CD/UC dataset. (TIF)
