# Supplementary figures and images for: Multi-omic modelling of inflammatory bowel disease with regularized canonical correlation analysis
Source: PLoS One. 2021 Feb 8;16(2):e0246367. doi: 10.1371/journal.pone.0246367 (PMC7870068; doi:10.1371/journal.pone.0246367)

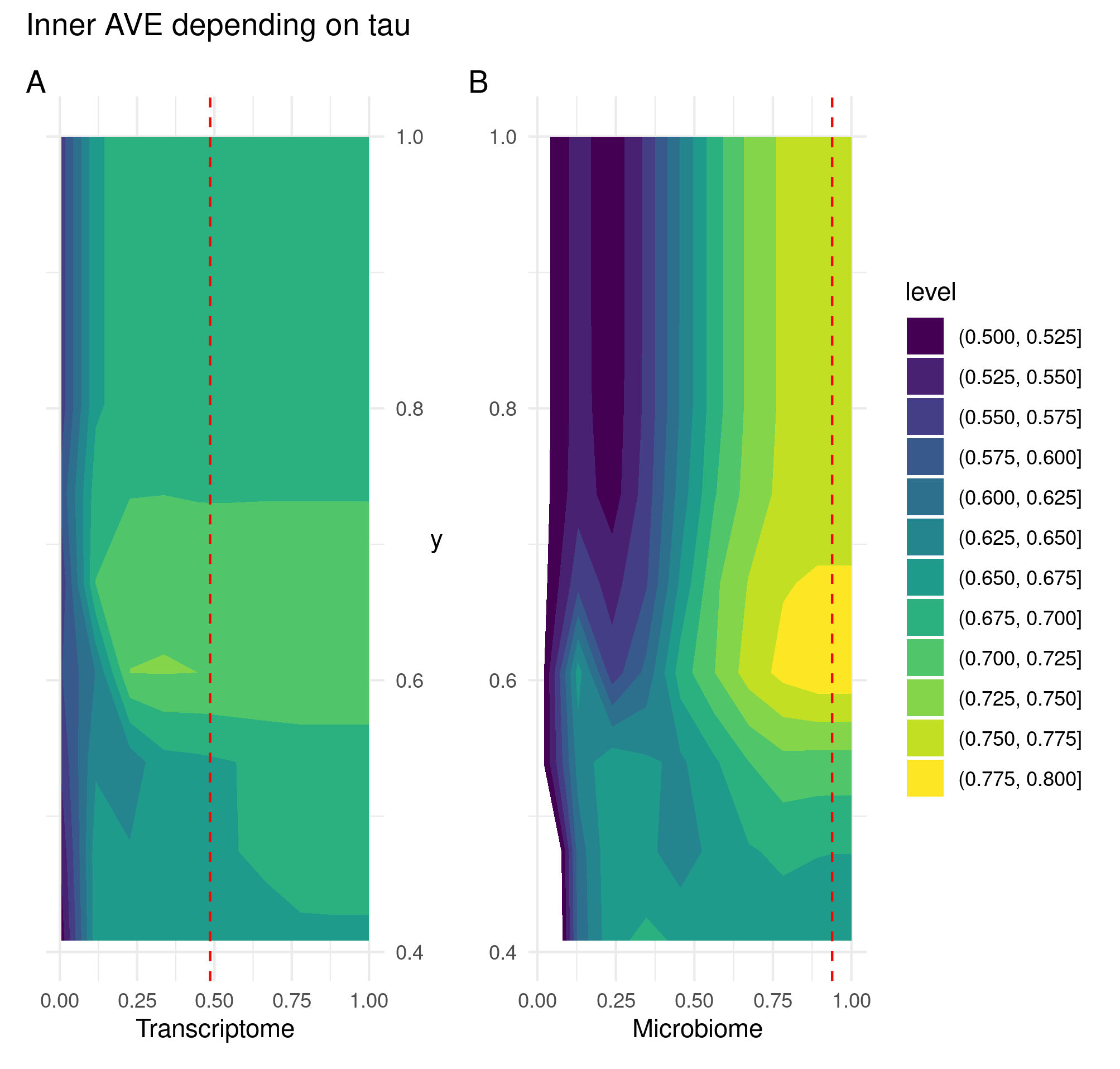

Supplement: S1 Fig — (TIF) [file pone.0246367.s004.tif]
